# Using infrared eye-tracking to explore ordinal numerical processing in toddlers with Fragile X Syndrome

**DOI:** 10.1186/1866-1955-5-1

**Published:** 2013-02-12

**Authors:** Emily R Owen, Heidi A Baumgartner, Susan M Rivera

**Affiliations:** 1 Department of Psychology, 1 Shields Avenue, University of California, Davis, CA 95616, USA; 2 University of California, Davis Center for Mind and Brain, 267 Cousteau Pl., Davis, CA 95618, USA; 3 M.I.N.D. Institute, University of California Davis Medical Center, 2825 50th St., Sacramento, CA 95817, USA

**Keywords:** Approximate number system, Development, Magnitude

## Abstract

**Background:**

Fragile X syndrome (FXS) is the most common cause of inherited intellectual disability and non-idiopathic autism. Individuals with FXS present with a behavioral phenotype of specific and selective deficits in an array of cognitive skills. Disruption of number processing and arithmetic abilities in higher-functioning adults and female adolescents with FXS has been well established. Still, both numerical skills and developmentally antecedent cognitive processes have just begun to be investigated in toddlers with FXS. The goal of the current study was to assess how very young children with FXS respond to ordinal relationships among numerical magnitudes.

**Methods:**

Infrared eye-tracking was used to explore infants’ novelty recognition during passive viewing of ordinal numerical sequences; *t*-tests were used to analyze group differences in looking time.

**Results:**

Ordinal recognition of numerical magnitudes is significantly impaired in young toddlers with FXS.

**Conclusions:**

This study is the first to experimentally evaluate early number sense and ordinal recognition in toddlers with FXS, and our findings reveal that ordinal recognition of numerical magnitudes is significantly impaired in young toddlers with FXS, suggesting that later arithmetic impairments associated with FXS may have their origins in a developmental impairment of this more basic aspect of numerical cognition.

## Background

Fragile X syndrome (FXS) is a neurodevelopmental disorder caused by a single-gene mutation on the X chromosome. FXS is the most common inherited cause of intellectual impairment, affecting approximately 1 in 3,600 males and 1 in 4,000 females [1]. It is also the most common cause of non-idiopathic autism [2,3]. FXS results from a trinucleotide (CGG) expansion on the 5’ untranslated promoter region of the fragile X mental retardation 1 gene (FMR1), located on a distal tail of the X chromosome [4]. The gene is normally polymorphic up to ~44 CGG repeats. The full FXS mutation is characterized by >200 repeats and renders the gene highly susceptible to DNA methylation and transcriptional silencing, which consequently results in a reduced level or complete loss of the gene’s protein product, fragile X mental retardation protein (FMRP) [5]. FMRP is an RNA binding protein involved in regulation of translation of multiple dendritic mRNAs important to synaptic development and plasticity, suggesting that FMRP is integral to the development of neural networks and functional integration across brain regions [6-8].

There is considerable evidence showing that cognitive deficits observed in individuals with FXS map onto abnormal processing in frontal-parietal neural networks in the brain. While the predominant end-phenotype of FXS is characterized by mild to severe intellectual impairment, individuals with FXS do not present with global deficits, but rather display selective impairments along with areas of spared skills. Individuals with FXS demonstrate marked weakness in performance on tasks of inhibitory control, selective and sustained attention, visual-spatial integration, motor coordination, and numerical processing [9]. The current body of literature has lead some researchers to suggest that abnormal connectivity between parietal cortex and occipital regions, as well as disruptions to a purported frontoparietal attentional network, underlie the specific cognitive profile seen in individuals with FXS [10-14]. Focusing primarily on the prominent cognitive deficit of mathematical disability observed in older and higher-functioning individuals with FXS, in this study we examine whether such impairments in the domain of numerical processing can be seen early in development.

Disruption of number processing and arithmetic abilities in higher-functioning adults and female adolescents with FXS is well established in the empirical literature [15-19]. In illustration, Rivera *et al.*[14] tested a group of adolescent girls with FXS on an arithmetic task and showed that they performed worse than controls, they also displayed a different pattern of brain activation when viewing simple arithmetic problems. Subjects exhibited less overall activation than did unaffected individuals during both 2-operand (e.g., 2 + 1 = 3) and 3-operand (e.g., 3 + 2 – 1 = 5) trials [14]. In response to increasing arithmetic complexity (i.e., going from 2- to 3-operand equations), unaffected subjects showed increased recruitment in parietal regions known to be associated with arithmetic processing (including left angular gyrus and intraparietal sulcus), whereas participants with FXS did not show this “ramping up” of parietal activation. Importantly, these researchers demonstrated that higher levels of serum FMRP were associated with more typical activation patterns in parietal areas known to support arithmetic processing [14]. Given the striking genetic dose-dependent nature of mathematical impairment seen in older individuals with FXS, the domain of numerical processing provides an important area for investigation of altered development and cognitive behavioral deficits in FXS. Still, neither mathematical skills nor developmentally antecedent cognitive processes (which presumably underlie more complex numerical processing), have been investigated in toddlers with FXS.

Research in typical cognitive development strongly suggests that human infants are able to represent and discriminate number at a very young age, long before verbal counting principles develop. A growing body of evidence supports the notion that human infants, adults and non-human primates all share a non-verbal system for representing quantity, and that this system is both ontogenetically primitive and is present very early in human development [20]. The approximate number system (ANS) [21] is theoretically and functionally distinct from that served by the verbally-based counting principles [22,23], and can be thought of as a mental “number line” which allows representation of imprecise magnitudes in terms of relative spatial relationships along a continuum [22,24]. The ANS is thought to support representation of specifically large, approximate numbers as non-exact, or “un-counted”, quantities, serving the judgment of “generally, how much”. Research in both adults and infants suggests that there is a distinct and separate system for representing small exact number, serving the judgment of “precisely one, two, three or four”. Evidence shows that the ANS and exact number systems develop at different rates [20,25]. In a task of numerical discrimination, Xu [25] demonstrated that typically developing 6-month old infants successfully discriminated between large numbers (4 *vs.* 8), but not small numbers (2 *vs.* 4), suggesting that ANS develops earlier than exact small number knowledge.

Studies of typically developing toddlers indicate that two-year-olds are able to recognize ordinal numerical relationships before they are able to use verbal counting to describe these relations, and Brannon [26] demonstrated that typically developing infants could detect ordinal relationships between numerical magnitudes by as young as 11 months of age. Implicit in the organization of the ANS is ordinality (the basic principle of which quantity comes first, second, third, etc.); the mental number line is ordered such that magnitude increases in one direction. Ordinality is also important for more exact numerical skills, such that stable order is essential to appropriate enumeration, development of verbal counting skills, and understanding of the relations among precise cardinal quantities represented by numerical symbols. Based on the “continuity hypothesis” [27], ontogenetically and evolutionarily primitive processes that support a general sense of quantity extend into basic cognitive representations of magnitude supported by the ANS, which developmentally precedes and underlies exact numerical representations. According to this view, perception of ordinal relationships is an essential skill for both approximate magnitude judgments and precise numerical processing and math abilities. Several researchers have proposed that perception of ordinal relations serves as the bridge across which exact numerical representations are mapped onto the more fundamental sense of approximate quantity [28,29]. In support of this perspective Halberda *et al.*[30], and Lyons and Beilock [29] demonstrated that individual differences in approximate magnitude judgments predicted symbolic math performance in adolescents and adults. Lyons and Beilock further demonstrated that among college students, better ability to order symbolic precise numbers significantly predicted performance on a mental arithmetic task, supporting the link between ordinal processing and more advanced mathematical cognition [29]. Nonetheless, these studies were conducted with relatively mature subjects and did not address developmental trajectories. It is therefore appropriate to examine ordinal processing in young individuals with FXS, who as a group, show deficits in later math ability that are considered hallmarks of the syndrome.

The goal of the current study was to examine the specific nature of mathematical deficits reported in individuals with FXS by investigating how very young children with FXS respond to ordinal relationships among numerical magnitudes. The tasks were adapted from Brannon [26] to address the early development of numerical knowledge in FXS and to begin to identify the developmental trajectory of math deficits displayed in older children and adults with FXS. This is the first empirical study of numerical processing in young toddlers with FXS, and results could shed light on the nature of math disability in FXS, as well as contribute to the developmental debate over the continuity of the ANS and mathematical skills. For example, if toddlers with FXS demonstrate impaired perception of ordinal magnitudes (as compared to typically developing (TD) developmentally age-matched controls), this would support the hypothesis that disruptions in the mental number line are associated with, and possibly underlie, dysfunction in higher-level math abilities in FXS. However, if toddlers with FXS demonstrate intact ordinal recognition, this would suggest that later deficits in precise number representation (e.g., arithmetic calculations) stem from qualitatively distinct processes in FXS.

## Methods

### Experiment 1

#### Participants

The research protocol (#297426-3) was approved by the institutional review board for human subjects at the University of California, Davis, USA. Sixteen toddlers (4 girls) with the FXS full mutation and sixteen developmental age-matched healthy TD toddlers (5 girls), were included in the final sample. Mean chronological ages for the FXS and TD groups were 35.85 months (range, 14.21 to 58.02 months) and 17.71 months (range, 12.02 to 21.11 months), respectively. Data from an additional six toddlers with FXS and four TD toddlers were not included because of insufficient gaze data in the familiarization trials or at least one pair of test trials.

TD toddlers were recruited by means of letters to families in Davis, CA, USA. Toddlers with FXS were recruited through the UC Davis M.I.N.D. Institute Fragile X Research and Treatment Center, where they were clinically evaluated and their diagnoses confirmed by molecular DNA testing.

To control for differences in development between the experimental groups, toddlers with FXS were assessed using the Mullen Scales of Early Learning (MSEL) [31] to determine developmental age. The mean developmental age in the FXS group was 19.24 months (range, 9.07 to 26.02 months), which matched well with that of participants in the TD group (17.71 months; range, 11.08 to 35.08 months). An independent-samples *t*-test confirmed that developmental level did not differ significantly between the two groups (*t*(30) = −1.12, *P* >0.20).

#### Apparatus

Toddlers’ gaze duration during the task was recorded with a Tobii 17-inch LCD binocular eye tracker. Stimuli were presented on the Tobii 1024 x 768 pixels resolution monitor, at a capture rate of 50-Hz, and a 60-Hz refresh rate. ClearView analysis software (version 2.7.1; Tobii Technology, Sweden) was used for calibration, allowing a best accuracy of 0.5 degrees.

#### Stimuli

**Figure 1:**
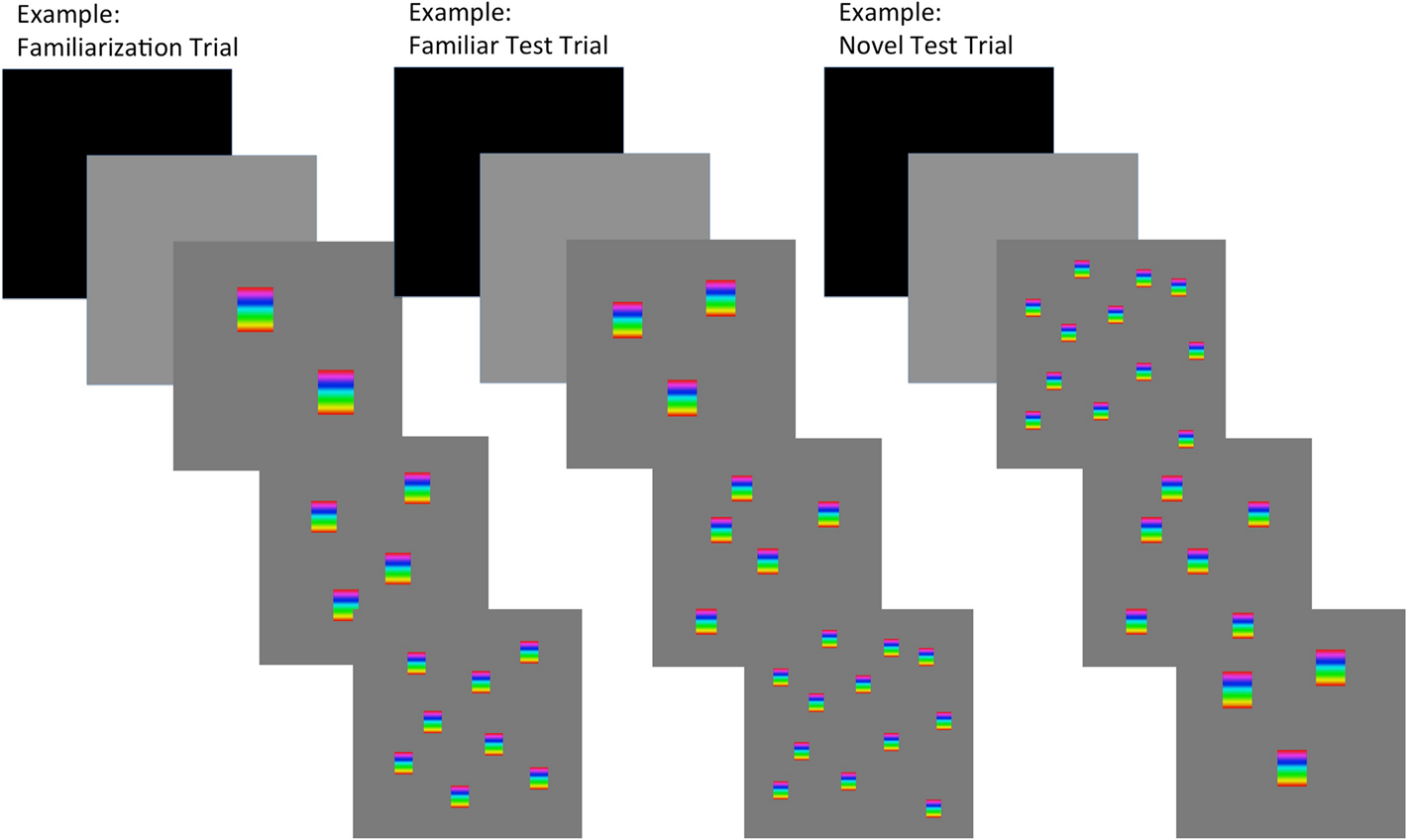
**Examples of the 5 frames of each trial type (for both experiments).** Each sequence of 5 frames repeated 3 times before the subsequent trial began.

Stimuli were created using Adobe Photoshop. Stimuli consisted of full-screen displays of different quantities of rainbow-colored squares presented in sets against a grey background. In accord with the ratios and numerical values used in Brannon [26], familiarization trials consisted of three sets of numerical sequences; the first set consisted of 2, 4 and 8 colored squares, the second 4, 8 and 16, and the third of 1, 2 and 4 squares. Test trials contained only one set of novel numerical values; 3, 6 and 12 squares (Figure 1). Cumulative surface area was kept constant across all numerical displays so that only number varied across sets and within sequences, and configuration of the squares in each display was randomly computer-generated as described previously [26].

#### Procedure

Informed consent was obtained from a parent of each participant before beginning testing. Toddlers were seated on a parent’s lap, 60 cm away from the eye-tracker monitor. The experimental session began with a five-point calibration module, followed by six familiarization and six test trials while audio sequences of classical music played from two standard computer speakers situated on either side of the monitor.

#### Design

In the interest of minimizing attentional demands on our FXS infant group, we chose to implement a familiarization paradigm rather than a strict habituation design. Toddlers were presented with a fixed familiarization phase of six trials, followed by the test phase of six trials. The familiarization phase consisted of either all ascending or all descending sequences of three numerical displays, while the test phase consisted of alternating ascending and descending sequences of novel numerosities (Figure 1). Half of the toddlers in each group were assigned to the ascending condition, and half to the descending condition. Each trial consisted of a five-frame cycle that was presented three times. The cycle began with a black screen followed by a grey screen and three sequentially-presented numerical values. After six full familiarization trials, toddlers saw six test trials in which ascending and descending sequences were presented in counterbalanced order with the novel direction, relative to familiarization, always presented first.

#### Familiarization trials

Familiarization trials begin with a black screen (250 ms) followed by a grey screen (250 ms), and then a sequence of three numerical values (1,000 ms each). This cycle was repeated three times so that a full trial consisted of three presentations of each numerical sequence and lasted 10.5 seconds. Since the experiment presented three different numerical sequences (2-4-8; 4-8-16; 1-2-4), twice each over the course of six familiarization trials, the entire familiarization phase lasted 63 seconds.

#### Test trials

Test trials were similar to familiarization trials except that they followed a numerically novel sequence (3-6-12) and alternated in their ordinal direction; i.e., ascending or descending numerical value. Thus, all trials were equally novel in numerosity compared to the familiarization trials, and the only net difference between test trials to which toddlers could respond was their ordinality. Each test trial began with a black screen (250 ms) followed by a grey screen (250 ms), and then presentations of 3, 6 and 12 squares in sequence (1 second each); this cycle was repeated three times so that a full trial lasted 10.5 seconds. Six test trials were presented, so this phase of the experiment also lasted a total of 63 seconds. Thus, it took just over two minutes to complete both phases of the experiment.

#### Coding

Eye tracking data were coded using the Area-of-Interest (AOI) definition tool within ClearView analysis software (Tobii Technologies, Sweden). AOIs were created by defining each frame (one full-screen numerical display) as an individual AOI (black and grey screens were not coded). The primary measure of interest was total looking time (the summed durations of all fixations) where a fixation was defined as gaze within the AOI for at least 200 ms. Coding began as soon as the frame appeared on screen and ended at the point of transition to the next frame (i.e., after 1,000 ms). For analysis purposes AOIs were grouped by numerical value and trial number, so that total looking time to all three displays of each numerosity were summed within a trial, and all looking times across numerical values were summed within a trial to provide the total cumulative looking time per trial.

We observed a lot of variance in participants’ overall looking duration to stimuli, both within and between groups. Therefore, for test trials, percent looking time was calculated for each participant by dividing their total looking time to novel and same trials respectively, by their total cumulative looking time to all test trials, to derive a measure of the proportion of each participant’s looking to novel *versus* familiar.

### Experiment 2

#### Participants

Infant participants in both FXS and TD groups were recruited in exactly the same manner as described for Experiment 1. Fourteen male toddlers with the FXS full mutation and twenty-one developmental age-matched healthy TD toddlers (5 girls) were included in the final sample. Six FXS toddlers (2 girls) were excluded from analysis due to insufficient looking time during the familiarization phase, associated with fussiness and excessive movement. Mean chronological ages for the FXS and TD groups were 32.13 months (range, 15.09 to 49.21 months) and 16.26 months (range, 12.22 to 21.11 months), respectively. The mean developmental age in the FXS group was 16.14 months (range, 12.00 to 21.08 months), which matched that of participants in the TD group (16.26 months; range, 12.22 to 21.11 months). An independent-samples *t*-test confirmed that developmental level did not differ significantly between the two groups (*t*(33) = 0.105, *P* >0.90).

#### Apparatus

All aspects of hardware apparatus are identical to that described in Experiment 1, except that participants were calibrated, and stimuli presented, through Tobii Studio software version 2.0.8 (Tobii Technologies, Sweden).

#### Stimuli

All aspects of stimuli are identical to those used in Experiment 1.

#### Procedure and design

All aspects of the overall procedure were identical to that in Experiment 1, except that to increase toddlers’ exposure to familiarization stimuli while still avoiding the greater attentional demands imposed by establishing habituation criteria, toddlers were presented with a familiarization phase of twelve trials, followed by the test phase of six trials. After twelve full familiarization trials, toddlers saw six test trials with ascending and descending sequences presented in counterbalanced order, with the novel direction relative to familiarization always presented first.

#### Familiarization trials

Trials were identical to those described in Experiment 1. As we extended the familiarization phase to twelve trials, the experiment presented three different numerical sequences (2-4-8; 4-8-16; 1-2-4) four times each and the entire familiarization phase constituted 126 seconds.

#### Test trials

Trials were identical to those described in Experiment 1.

#### Coding

Eye tracking data was coded using the AOI definition tool within Tobii Studio. Looking times were calculated for test trials in the manner described for Experiment 1.

## Results

### Experiment 1

A Kolmogorov-Smirnov test was run to check the distribution for the percent looking time variable and it was insignificant for both groups (TD: D(15) = 0.942, *P* = 0.338); FXS: D(15) = 0.792, *P* = 0.558), indicating that our data were normally distributed and therefore that parametric tests were appropriate. Preliminary analyses revealed no significant differences between ascending or descending conditions of the task, nor between males or females, so data from both conditions and genders were collapsed within experimental groups to increase power for subsequent analyses.

**Figure 2:**
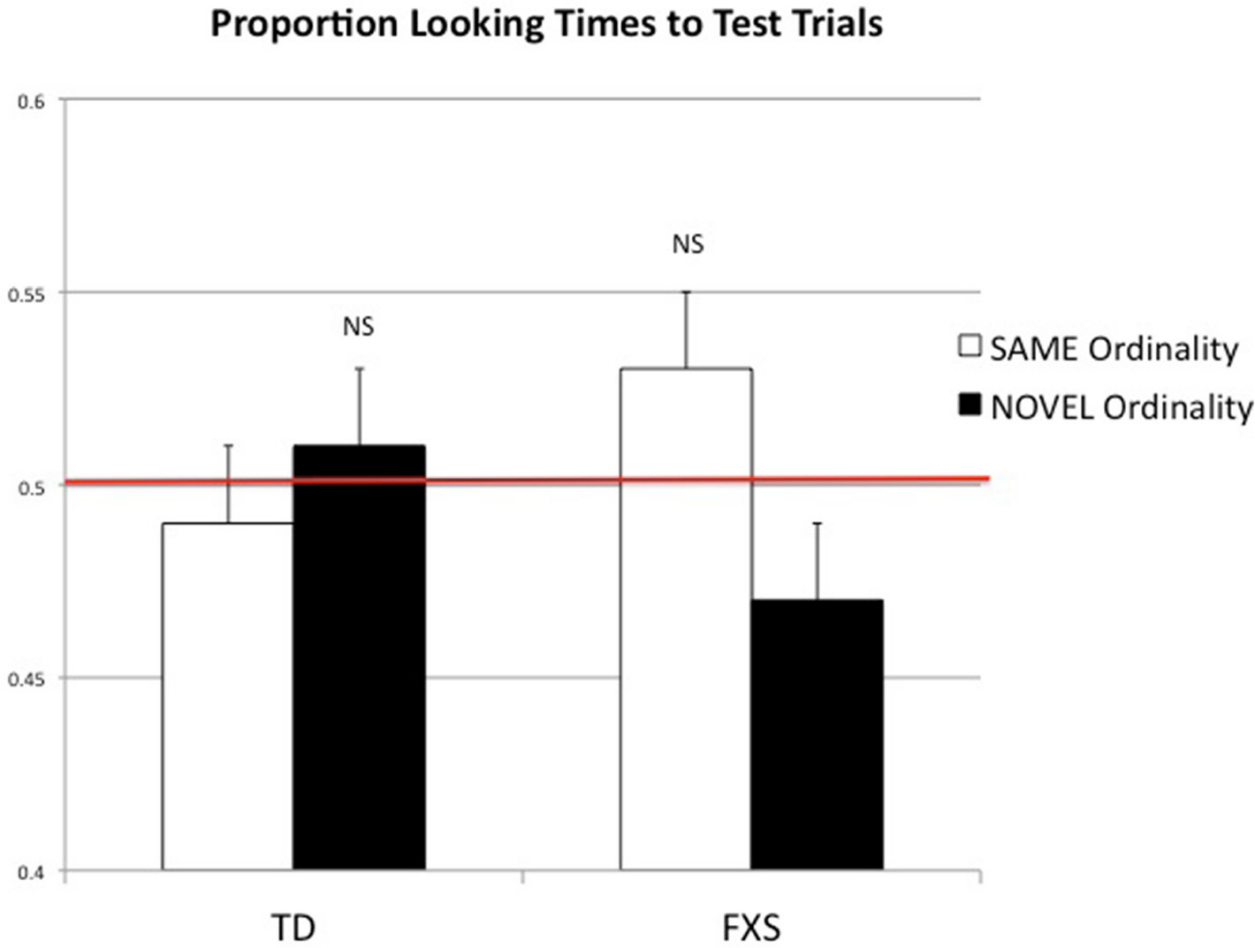
**Group proportion looking time (ms) to test trials (Experiment 1).** Average percent looking time to novel and same test trials in TD and FXS groups; both groups of toddlers showed no preference above chance (TD: *t*(15) = ±0.307, *P* = 0.763; FXS: (*t*(15) = ±1.250, *P* = 0.230).

TD toddlers and toddlers with FXS did not display different overall looking behavior during the test phase trials (Figure 2). We conducted one-sample *t*-tests for each group, comparing percent looking times to test trials to chance (50%). Independent *t*-tests confirmed that looking times to both types of test trials were statistically equivalent to chance (*t*(15) = 0.307, *P* = 0.763). FXS toddlers spent a larger proportion of their looking time on the familiar test trials than the novel trials (47% to novel, 53% to familiar), but independent *t*-tests revealed that these looking times were also not significantly different from chance (*t*(15) = 1.250, *P* = 0.230). Thus, the FXS group trended towards a familiarity preference, and both groups failed to detect the novel ordinal relationship. We also analyzed the FXS data with just the male toddlers, taking out the four females who may, because of their one normally functioning X chromosome, have performed differently from the males. Without females in the analysis the males with FXS showed a slightly stronger trend towards a familiarity preference, but this difference still failed to reach significance (*t*(11) = 1.860, *P* = 0.088).

In our sample TD and FXS toddlers displayed no preference in their looking behavior for either same or novel trials. To investigate the level to which participants in both groups were familiarized to the stimuli in the familiarization phase, we examined looking behavior during the familiarization phase and found that paired *t*-tests indicate a significant decrease in the total looking time between the first three and last three familiarization trials (16.79 s and 13.16 s, respectively) for the TD group (*t*(15) = 2.78, *P* = 0.014, 2-tailed). However, while the FXS group displayed a trend in this direction, the difference in total looking duration between the first three and last three familiarization trials (13.59 s and 10.47 s, respectively) failed to reach significance (*t*(15) = 1.89, *P* = 0.078, 2-tailed).

### Experiment 2

As for Experiment 1, the Kolmogorov-Smirnov test for goodness of fit was insignificant (TD: D(20) = 0.363, *P* = 0.999; FXS: D(13)= 0.562, *P* = 0.910), thus parametric tests were applied to the data. A comparative analysis revealed no significant differences between ascending or descending conditions of the task, so data from both conditions (and gender for the TDs) were collapsed within experimental groups to increase power for subsequent analyses.

**Figure 3:**
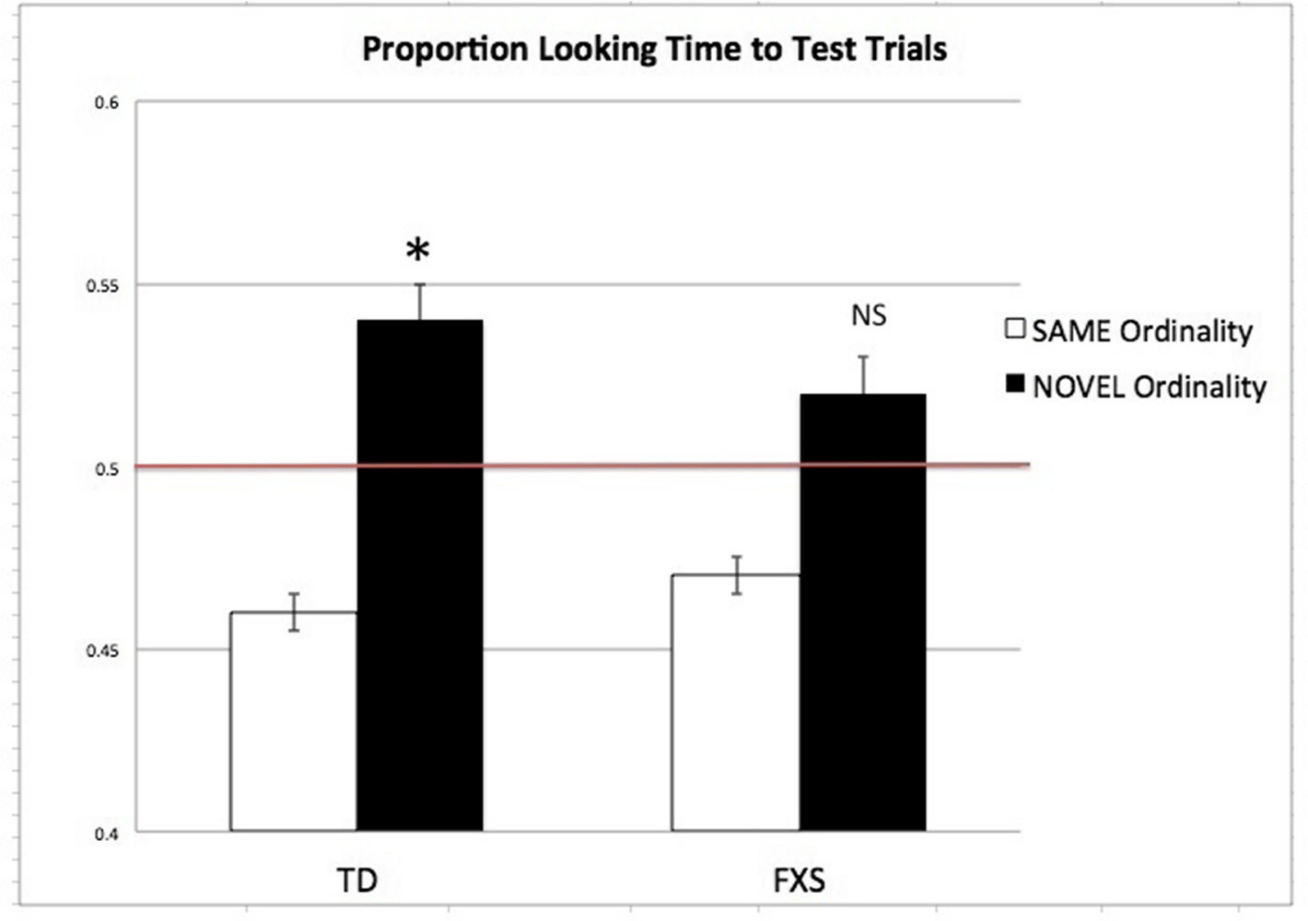
**Group proportion looking time (ms) to test trials (Experiment 1).** Average percent looking time to novel and same test trials in TD and FXS groups; both groups of toddlers showed no preference above chance (TD: *t*(15) = ±0.307, *P* = 0.763; FXS: (*t*(15) = ±1.250, *P* = 0.230).

Paired *t*-tests of total looking duration indicated that both groups significantly decreased their total looking time from the first three (TD group mean = 20.50 s; FXS group mean = 22.62 s) to the last three familiarization trials (TD group mean = 15.60 s; FXS group mean = 18.01 s), (TD group: *t*(20) = 4.08, *P* <0.01, 2-tailed; FXS group: *t*(13) = 4.80, *P* <0.01, 2-tailed). Percent looking times to novel test trials were entered into one-sample *t*-tests against 0.50, as for Experiment 1 (Figure 3). Independent *t*-tests revealed that these looking times were statistically different from chance for the TD group only (*t*(20) = 2.661, *P* = 0.01). FXS toddlers’ proportion of looking time to novel and test stimuli were not significantly different from chance (*t*(13) = 1.806, *P* = 0.09). The results from Experiment 2 indicate that toddlers with FXS differ significantly from developmentally-matched TD toddlers in their ability to detect the ordinality of a sequence of numerical magnitudes. Results from this experiment replicate findings from Brannon [26]; specifically, that recognition of numerical magnitude is a developmentally early skill typically present by the first year of life. Importantly, our results extend these findings to young individuals with FXS, demonstrating that an early disruption of ordinal perception exists in the fragile X syndrome, and suggesting an association with the deficits in numerical processing and math abilities seen in older individuals with FXS.

## Discussion

### Experiment 1

Brannon notes that in the context of her strict habituation paradigm, 11-month old toddlers required an average of eight trials to meet habituation threshold [26]. In this experiment we only presented six trials, and the toddlers saw the stimuli for much less time, in total, than did the toddlers in Brannon’s study [26]. Based on the existing literature regarding infant looking behavior we reasoned that our experimental design did not allow enough stimulus exposure for toddlers in either group to fully familiarize to the initial ordinal pattern. Infant looking behavior follows a general trajectory throughout the process of stimulus habituation [32]. Toddlers’ preference for novel *versus* familiar stimuli progressively develops over the course of familiarization, so that toddlers who are not fully familiarized prefer familiar stimuli, but reliably demonstrate a preference for novelty once fully familiarized [33,34]. Our results suggest that neither group of toddlers were fully familiarized to the stimuli. Experiment 2 was designed to test our hypothesis that toddlers require more exposure to stimuli in order to become familiarized to ordinal sequences in this particular experimental design.

### Experiment 2

The experiments reported here were designed to investigate early number sense and ordinal numerical representation in very young individuals with FXS, in the context of exploring the developmental trajectory of mathematical deficits in FXS. Results from Experiment 2 suggest that within an extended familiarization paradigm, toddlers with FXS are unable to appropriately represent and recognize ordinal numerical sequences. These results suggest that the marked deficits in math ability and numerical processing seen in older individuals with FXS might stem from a failure to recognize ordinal relations.

## Conclusions

The experiments reported here were designed to investigate early number sense and ordinal numerical representation in very young individuals with FXS. Results from Experiment 2 suggest that toddlers with FXS are unable to represent and recognize ordinal relations in comparison to typically developing toddlers. Still, in order to further elucidate the precise nature of the numerical processing deficits seen in FXS, we will need to directly test several different aspects of number sense in young individuals with FXS.

Additionally, it is important to address some limitations of the current study. First, it is important to note that while we did control for overall cumulative surface area of our numerical displays (as did Brannon in her original 2002 task [26]), the spatial density of the different numerosities did inevitably vary (e.g., the “2” display took up the same amount of space as the “8” display, but 2 squares cannot be as distributed around the screen as can 8 squares). However, given the well-established visuospatial processing differences seen in individuals with FXS, one would not necessarily expect this group to be able to use this information to detect ordinality, and indeed they did not in our task.

Perhaps most important to address is the issue that the stimuli used in this task spanned numerosities that are typically considered to be served by the two distinct number subsystems; approximate and exact. Specifically, the exact number system deals with differences between 1, 2 or 3 items, while the approximate system serves the ability to discriminate between large, un-counted quantities. Our familiarization stimuli consisted of one sequence of all small number (1-2-4), all large number (4-8-16), and a mix of large and small (2-4-8). The test stimuli presented a mix as well (3-6-12). Still, we used the exact same numerical sequences as Brannon [26], and replicated her findings in TD infants. Moreover, we looked at the cumulative looking time to the different sequences and found no differences in either our FXS or TD group in terms of how long they looked at small, mixed or large sequences, nor to individual small or large number displays (for example, display of ‘2’ *vs.* display of ‘8’) (Table 1). That said, with the present data we cannot rule out that a prevailing deficit, or developmental delay, in one or both of these subsystems is responsible for the failure of the FXS group to detect the overall ordinality of the sequences.

**Table 1:** Average looking time to small, mixed and large numerosity sequences (Experiment 1)

	**1-2-4 (small)**	**2-4-8 (mixed)**	**4-8-16 (large)**
**TD**	7782 ms	9353 ms	7724 ms
**FXS**	5990 ms	6695 ms	6540 ms

Average looking time per sequence type for both groups. There were no statistically significant differences between looking duration to small, mixed or large numerosities for either group.

To address this possibility, it would be necessary to further test toddlers with FXS on two similar but diametrically opposed tasks; one involving only small numbers, and the other only large, approximate numerosities. While we support this approach to further elucidate how toddlers with FXS deal with small and large numbers, we can foresee a few issues with the interpretation of differential performance within these ostensibly distinct subsystems. First, most research on exact and approximate numerical processing that supports separate subsystems in infants and toddlers employs simple discrimination paradigms, which we argue are qualitatively different from this task. In a discrimination task one is required to make a “less than” or “more than” judgment, whereas in this task ordinal relationships must be detected by a rank order judgment (such as “before” or “after”), which at least intuitively maps onto the ‘number line’ concept of the ANS. It is certainly possible that in this context small and large numerosities are not processed so differently, but rather according to a common process of relational associations [29,35]. Finally, our toddlers are both developmentally and chronologically much older than infants who, when tested in laboratory experiments, showed differential performance on small and large number tasks [25], thus in toddlers of this age, even if they do have delays, we may not be taxing any one system more than the other.

More importantly we believe caution must be taken in making the assumption that there are necessarily two separate and neurally differentiated number subsystems operating in toddlers with FXS. Rivera *et al.* tested typically-developing school-age children through adulthood and demonstrated a developmental trajectory of increasing functional specialization in the inferior parietal lobe for arithmetic reasoning [36]. Taking a neuroconstructivist perspective, this pattern of development suggests that neural subsystems identified later in development might not be innately pre-specified, but rather reflect experienced-based developmental trajectories from a relatively domain-general infant brain towards differentiated and specialized adult circuitry. In illustration of this point, a recent paper by Karmiloff-Smith and colleagues discusses the problem with applying a modular approach to understanding developmental syndromes [37]. They report performance differences between infants/toddlers with Down Syndrome (DS) and Williams Syndrome (WS); the DS group showed impaired discrimination between small numbers but not large, while the WS group had trouble discriminating large approximate numbers but performed equal to TDs on a small number discrimination task. They interpret these findings not as evidence for different patterns of domain-specific deficits *versus* intact subsystems, but rather in terms of domain-general visual attention problems specific to each syndrome that likely have downstream consequences across multiple cognitive domains, including the visual processing of numerical displays. We favor this kind of interpretation of numerical processing in FXS as well. Indeed, there is ample evidence that FXS involves abnormal visual attention in infancy [11,38], and our lab has shown marked deficits in aspects of spatiotemporal processing and temporal attention in infants with FXS much younger than the toddlers tested in this paper [10,39]. Thus, while some researchers have reported separable subsystems for large and small number processing in typically developing infants [25], we consider that early differences in attentional development in FXS may well have affected the development and organization of neural circuitry supporting other cognitive processes such as number processing. It is therefore intriguing and developmentally parsimonious to consider that the very early visual attention deficits associated with FXS have significant cascading effects that result in the distributed and seemingly selective pattern of cognitive strengths and weaknesses characteristic of older individuals with the syndrome.

Thus, the experiments reported here do not necessarily allow for a conclusion about purely numerical processing *per se* in young individuals with FXS, but rather support the notion that more basic disruption in visual attention affects the development of other cognitive processes. To meaningfully examine cascading effects we would have to move away from cross-sectional methodology and undertake longitudinal investigations. Indeed, longitudinal approaches to the study of development are arguably most essential when investigating the neural change over time and consequent alternative developmental pathways inherent in developmental disorders such as FXS. Encouragingly, recent longitudinal studies of young people with FXS reveal that early attention is a predictor of later cognitive and behavioral outcomes [40-42], and that physiological measures of arousal in toddlerhood predicts autistic symptomology in childhood [43]. The results from these studies bolster our speculation that early attentional capabilities drive the developmental trajectories of specific cognitive and behavioral outcomes, and moreover provide a strong endorsement for the fruits of longitudinal labor in understanding dynamic change in developmental disorders.

In conclusion, despite the limitations discussed above, we have shown that toddlers with FXS failed to discriminate the ordinality of a numerical sequence, while developmentally-matched TD controls did recognize ordinality in this task. This is one of the first studies to investigate very early numerical processing in FXS and uniquely informs on the existing body of research on mathematical abilities in older individuals with FXS. In particular, we are encouraged that taking a longitudinal dynamic developmental approach to studying the end phenotype of the syndrome lends itself to the development of early, syndrome-specific and domain-general interventions that are better positioned to effect broad-based cognitive outcomes. In the future, we plan to investigate non-numerical ordinal processing, as well as to compare large approximate and small exact numerical recognition, to more directly address this question of number sense in toddlers with FXS. Most importantly our future projects will employ longitudinal methodology, and do so within a theoretical framework of visuoattentional processing, to better understand the nature of developmental change and outcomes in FXS.

## Abbreviations

AOI: Area of interest; ANS: Approximate number system; FMRP: Fragile X mental retardation protein; FXS: Fragile X syndrome; MSEL: Mullen scales of early learning; TD: Typically developing.

## Competing interests

None of the authors have any financial or non-financial competing interests.

## Authors’ contributions

EO participated in making stimuli and conceiving of and designing Experiment 2 of the study. EO also participated in data acquisition, performed all statistical analyses, and data interpretation. EO drafted the manuscript. HB participated in making stimuli for, designing and carrying out pilot administration of Experiment 1 of the study, and helped to revise the content of the manuscript. SR made substantial contributions to study conception and design, participated in manuscript revision, and approved the final version of this manuscript. All authors read and approved the final manuscript.
